# PARP Inhibitors in Combination with Radiotherapy: To Do or Not to Do?

**DOI:** 10.3390/cancers13215380

**Published:** 2021-10-27

**Authors:** Amelia Barcellini, Pierre Loap, Kazutoshi Murata, Riccardo Villa, Youlia Kirova, Noriyuki Okonogi, Ester Orlandi

**Affiliations:** 1 Radiation Oncology Clinical Department, National Center for Oncological Hadrontherapy (CNAO), 27100 Pavia, Italy; amelia.barcellini@cnao.it (A.B.); riccardo.villa@cnao.it (R.V.); ester.orlandi@cnao.it (E.O.); 2 Department of Radiation Oncology, Institut Curie, 75005 Paris, France; Youlia.kirova@curie.fr; 3 National Institutes for Quantum and Radiological Science and Technology, QST Hospital, Chiba 263-0024, Japan; murata.kazutoshi@qst.go.jp (K.M.); okonogi.noriyuki@qst.go.jp (N.O.)

**Keywords:** BRCA, PARP-I, Poly-ADP ribose polymerase, radiotherapy, toxicity, synthetic lethality

## Abstract

**Simple Summary:**

Despite the large use of inhibitors of Poly-ADP ribose polymerase (PARP-I), the feasibility and safety of their combination with radiotherapy (RT) are unclear. The combination may be particularly interesting in the oligometastatic setting in which patients may benefit from local RT during the treatment with PARP-I. The aim of the current review was to evaluate the outcome and the toxicity in patients with newly diagnosed or recurrent tumors treated with a combination of PARP-I and RT. A total of 12 clinical studies met the inclusion criteria and, despite the heterogeneity of the evaluated patient populations and tumor types, this review suggests that a combination approach is feasible even though the efficacy profile remains unclear.

**Abstract:**

Background: Despite the large use of inhibitors of Poly-ADP ribose polymerase (PARP-I), the feasibility and safety of their combination with radiotherapy (RT) is unclear. Aim: We conducted a literature analysis with the aim to evaluate the efficacy and safety profile of a combination with RT and PARP-I. Method: The key issues for the current review were expressed in two questions according to the Population, Intervention, Control, Outcome (PICO) criteria: 1. What is the outcome and 2. What is the toxicity in patients treated with a combination of PARP-I and RT for a newly diagnosed or recurrent tumors? Results: A total of 12 clinical studies met the inclusion criteria including seven single-arm dose-escalation phase I studies, two phase II (two- and three-arms controlled trials) trials, one parallel-arm phase I study, and two phase I/II studies published between 2015 and 2021. RT was performed with photon beams and several schedules according to the clinical situation. The acute toxicity ≥ grade 3 ranged between 25% and >96%, which was divided into hematological or non-hematological adverse events. Conclusions: despite the heterogeneity of the evaluated patient populations and tumor types, and the limited number of the studies, this review suggests that a combination approach is feasible even though the efficacy profile remains unclear.

## 1. Introduction

Poly-ADP-ribose polymerase inhibitors (PARP-I) are able to block the Base Excision Repair (BER) mechanism, impeding the reparation of single-strand breaks (SSB), and, consequently, leading to the formation of double-strand breaks (DSB) causing the collapse of the replication fork [1,2]. Several tumor lines have developed defects and mutations in proteins involved in the control and repair of DNA damage, such as p53, ATM, MRE11 and BRCA1-2 [1,2,3]. Tumor cells with the above-reported mutations, if treated with PARP-I, are more sensitivity to cytotoxic chemotherapic agents [1,4,5] and this synthetic lethality when combined with ionizing radiation is p53 dependent [6]. Generally, PARP-1 (a member of the family PARP enzymes, which plays an important role as DNA discontinuity sensors as well as in SSB repair BER) does not directly contribute to the repair of DSB, but when DSB reparation mechanisms are deficient, such as the homologous recombination (HR) pathway, PARP-1 inhibition can lead to cell death [2,7]. This process is called synthetic lethality and is one of the most thrilling indicators of progress in oncology in recent years. For their action on DNA, PARP-I act as radiosensitizing agents and this is the reason why the combination approach between PARP-I and radiotherapy (RT) has been explored on several cell lines [8]. Even if several trials are conducted or are still ongoing about the combination approach (RT + PARP-I) in cancer patients, there is a lack of knowledge about the toxicity profile and the correct sequence of administration of these treatments (sequential vs. sandwich vs. concomitant) is still unclear. For this reason, we conducted a literature analysis to evaluate the efficacy and safety profile of a combination with RT and PARP-I.

## 2. Material and Methods

### 2.1. Search Strategy

**Figure 1:**
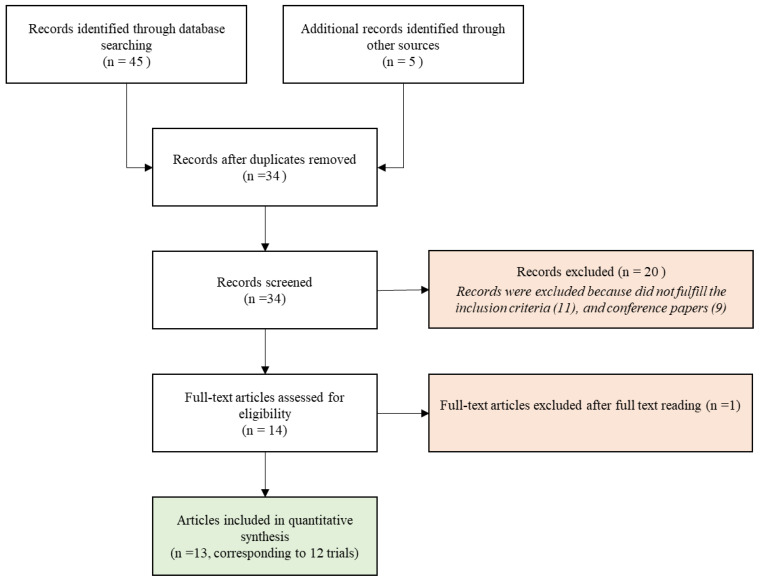
PRISMA Flow diagram of the study selection process.

According to the Population, Intervention, Control, Outcome (PICO) method [9,10], we defined two research questions (Table 1) that were the cornerstone of literature research in the main databases (PubMed, Web of Science, Google Scholar and Scopus) through the ensuing matched keywords: “Parp-inhibitor”, “PARP-I”, “Poly-ADP ribose polymerase”, (also including “Olaparib”, “Niraparib”, “Rucaparib”, ”Talozaparib”) “Radiotherapy”, “Radiation”, “Hadrontherapy”, “Radiosensitizer”, “Synthetic Lethality”, “Toxicity”, “Stereotactic radiotherapy”, pluralization and US English/UK English spelling variations and suffixes/prefixes. In July 2021, we conducted a search using the Preferred Reporting Items for Systematic Reviews and Meta-Analyses (PRISMA) literature selection process (Figure 1) [11]. Two authors (AB and PL) independently performed the literature search.

### 2.2. Selection Criteria for Full-Text Article Review

A full-text review was performed to include the publications that met the following criteria: (1) full article in peer-reviewed journals; (2) concomitant RT (photon beam RT, hadrontherapy, brachytherapy, intraoperative RT, electron beam RT) with PARP-I; (3) clinical studies; (4) at least one of the analyzed outcomes (toxicity and outcome); (5) articles written in the English language. Interventional, observational, prospective, and retrospective studies can be considered. Exclusion criteria were: (1) sequential treatments (RT followed by PARP-I or vice versa without concomitant combination); (2) presentation of clinical trials without data; (3) single case reports, book chapters, books, or conference proceedings.

## 3. Results

A total of 13 publications met the selection criteria. Overall, 12 clinical studies were eligible for analysis considering that two publications [12,13] independently reported acute and late toxicities of the same trial. The selection of studies analyzed in the present review is shown in Figure 1.

Table 2 showed the study characteristics and Table 3 the outcomes of the selected studies separating the studies evaluating the combination of RT with PARP-I (double combination, Table 3a) from those testing a triple combination (RT + PARP-I + other drugs, Table 3b).

### 3.1. Population

Seven single-arm dose-escalation phase I studies, one parallel-arm phase I, two phase II (two- and three-arms controlled trials) trials, and two phase I/II studies published between 2015 and 2021 were included.

A total of 823 patients with an age ranging between 2.2 and 86 years old who underwent concomitant radiotherapy and PARP-I administrations were analyzed.

Overall, 388 patients (47.1%) were enrolled in trials evaluating treatment combination for brain metastases, 125 (15.2%) for glioblastomas, 80 (9.7%) for non-small-cell lung carcinoma (NSCLC), 30 (3.7%) for pancreatic cancers, 32 (3.9%) rectal cancers, 32 (3.9%) for gynecological tumors, 54 (6.6%) for breast cancers, 16 (1.9%) for head and neck localizations and 66 (8%) pediatric patients for pontine gliomas. Overall, 667 patients (81.04%) of all cases received PARP-I in these trials (and not placebo or standard therapy).

### 3.2. Intervention

RT was performed with photon beams and several schedules according to the clinical situation. In 755 patients (92% of all patients enrolled in the current analysis), the analyzed PARP-I was Veliparib [14,15,16,17,18,20,21,22,23,24]. Olaparib was administered for triple-negative breast cancers (24 patients, 3%) [12,13], NSCLC (28 patients, 3.4%) [22] and locally advanced head and neck cancers (16 patients, 2%) [19]. In the series by Baxter et al. [21] and Sim et al. [24] the patients underwent concomitant Temozolomide according to the histology; in locally advanced pancreatic cancer concomitant Gemcitabine 400 mg/m^2^ was prescribed [20] and Capecitabine 825 mg/m^2^ twice daily was administered in rectal tumors [16]. Paclitaxel 45 mg/m^2^ and Carboplatin (CBDCA) area under the curve (AUC) 2 weekly concomitant and in consolidation were delivered in NSCLC series [23]; Cetuximab was administered starting approximately 5 days before RT in Karam et al. [19]. In the phase I study by de Haan et al. [22] patients with unresectable loco-regional or oligorecurrent NSCLC were treated in two parallel-arms, one of which with concomitant daily Cisplatin (CDDP) 6 mg/m^2^.

### 3.3. Comparison

A direct comparison between RT+ PARP-I and RT + Placebo/Standard Treatment was performed in three studies [15,23,24]. In one study [22], patients were treated in two parallel arms with or without concomitant chemotherapy.

Chabot et al. randomized patients with brain metastases from NSCLC to whole brain RT (Total dose: 30 Gy over 10 fractions) + Veliparib (50 mg) twice daily (BID; *n* = 103), Veliparib BID (200 mg; *n* = 102), or placebo (*n* = 102) [15].

In the phase I part of the trial by Argiris et al. [23], patients with stage III NSCLC were treated with three different doses (40, 80, and 120 mg) of Veliparib BID during chemo-radiotherapy (ChemoRT). Chemotherapy was administered once weekly with Paclitaxel 45 mg/m^2^ and Carboplatin AUC 2. In this first phase, two of the 21 enrolled patients experienced dose-limiting toxicity (DLT) at 40 mg (esophagitis leading to dysphagia) and 80 mg (esophagitis leading to dehydration) but not at 120 mg BID that was the recommended dose for a subsequent phase II trials on 31 patients. The design of this phase II study was: Veliparib + ChemoRT vs. Placebo + ChemoRT followed by consolidation with chemotherapy (Paclitaxel and Carboplatin) + Veliparib or Placebo.

Besides, VERTU trial [24] randomized 2:1 glioblastoma patients to Stupp regimen vs. Veliparib 200 mg BID + RT followed by the combination of Veliparib 40 mg BID days 1–7 and Temozolomide 150–200 mg/m^2^ OD (once a day) days 1–5, repeated every 28 days for 6 months.

Finally, De Haan et al. [22], who tested Olaparib in a dose-escalation trial, separated patients in two parallel arms: concomitant RT-PARP-I with daily low dose CDDP (6 mg/m^2^) vs. RT-PARP-I only.

### 3.4. Outcomes

In Table 3a,b, treatment-related toxicities and oncological outcomes of the analyzed studies are summarized.

Considering the first research question, endpoints (LC, OS and PFS) were calculated using different criteria. Data on median OS were available in three studies and ranged between 9.1 and 14.6 months, while the median PFS ranged between 3.6 and 9.8 months [17,20,24]. Considering the different histologies, tumor stages, and the related prognoses, LC, PFS and OS were different in the analyzed studies, as shown in Table 3a,b, and difficult to objectively compare. However, in the three trials in which comparison arms were available, the differences can be summarized as follows:

- In the first trial by Chabot et al. [15] no differences in survival rates or toxicity across the arms were found;
- In the phase I part of the trial by Argiris et al. [23], even if the early closure of the study did not allow to evaluate the full efficacy of the combination of Veliparib, the PFS and the OS from registration to consolidation were not statistically different between the ChemoRT arm and the ChemoRT + Veliparib arm.
- In the VERTU trial, no significant clinical benefit was found (Median PFS of 5.7 months (95% CI: 3.9–6.5 months) in the experimental arm vs. 4.2 months (95% CI: 2.4–5.7 months) in the standard arm) [24].

Among the considered studies, the acute toxicity ≥ grade 3 ranged between 25% and > 96%, which can be further divided into hematological or non-hematological adverse events.

Concerning hematological grade ≥ 3 toxicities, lymphopenia ranged between 3% [16] and 96% [20] with febrile neutropenia described in 4% of the Tuli’s series [20]; anemia was experienced incidence ranged between 3% [16] and 38% [20], and thrombocytopenia occurred in up to 23.1% of the cases [21]. Jagsi et al. [18] did not describe late grade ≥ 3 hematological toxicities. The combination usually appeared safe with regards to hematological toxicity both in double and in triple combinations (lymphopenia ranged between 5–59% vs. 3–96%; thrombocytopenia between 2–12% vs. 3–22.1% and anemia 1–9% vs. 3–38%, respectively). Moreover, in the update of the RADIOPARP [13], no treatment-related grade ≥ 3 toxicities were reported even if the authors described a case of grade 4 thrombocytopenia experienced by a woman subsequently treated with systemic therapy in a metastatic setting.

In the above-reported studies with an available comparison arm:

- Chabot et al. [15] did not describe a significant difference in toxicity across the arms, even if a lower incidence for Grade 3/4 adverse events in the Veliparib arms (50 mg versus 200 mg; *p* < 0.05) was reported.
- In the phase I part of the trial of Argiris et al. [23], 19% of patients underwent grade 4 toxicities, which included lymphopenia (3 cases) and neutropenia (1 case) and 57% showed grade 3 adverse events (AEs), which were mostly hematologic and not associated with dose levels. In the phase II part of the trial, the authors reported 18% of neutropenia and 3% of thrombocytopenia.
- Veliparib-containing regimen was well tolerated in the VERTU trial [24], with thrombocytopenia and neutropenia, corresponding to the main grade ≥ 3 toxicities, being more frequent in the experimental arm (12% vs. 3% and 17% vs. 8%, respectively).
- In the parallel-arm trial by de Haan et al. [22] grade ≥ 3 acute toxicities increased from 45% (Olaparib 25 mg once daily), 57% (Olaparib 25 mg twice daily) to 80% (CDDP + Olaparib), and hematologic grade ≥ 3 toxicities other than lymphocytopenia were only observed in patients treated with concomitant CDDP.

With regards to non-hematological grade ≥ 3 toxicities, gastrointestinal adverse events were common with nausea, diarrhea and colitis being the most frequent. Argiris et al. described a case of esophageal perforation leading to treatment-related death 8 months after the end of the treatment in phase I part of the study [23]. Other common adverse events were fatigue (from 4% to 10%) [14,15,17,20,23], hyperglycemia (3–4%) [14,15], electrolytic imbalance (hyponatremia [14], hypomagnesemia [19] and dehydration [19]). Czito et al. [16] reported also vaginal stricture (3%) and post-actinic enteritis (3%) for rectal cancer treatment, as well as lung embolism (3%) which was similarly observed by Chabot et al. [15] (1% for the placebo arm, 4% for Veliparib 50 mg and 2% for Veliparib 200 mg). Fibrosis was reported by Jagsi et al. [18] up to 40% of the patients at 3 years in addition to skin induration (13%). Karam et al. [19] highlighted dermatitis (38%) for head and neck patients treated with Olaparib, which was also reported by Loap et al. [12] up to 8% for breast cancer patients undergoing Olaparib. However, in the 1-year analysis, the same group [13] did not describe any treatment-related grade ≥3 late toxicity. In diffuse pontine gliomas, maculopapular rash (3%) and neurologic deterioration (3%) were described [21]. For De Haan et al. [22] the incidence and severity of late esophageal toxicities decreased in patients treated without CDDP and with a lower Olaparib dose and, even if authors reported 18% grade 5 pulmonary side effects, from an exploratory analysis these severe toxicities appear to be related to the RT lung dose.

## 4. Bias and Limitation of the Analysis

The major limitations of the current analysis that do not allow to draw definitive conclusions were: the heterogeneity of evaluated patient populations and tumor types, the usually small-sized cohorts, the shorter follow-up and the absence, for most of the clinical studies, of comparison arms.

Moreover, not all the studies were dose-escalation trials and for this reason it was not possible to draw definitive conclusions about the maximum tolerated dose (MTD) in each setting.

Besides, the several clinical settings analyzed in the current review implied different RT volumes, doses and treatment fields that are pivotal factors to be considered in the assessment of loco-regional toxicities.

Moreover, Veliparib, the main PARP-I reviewed, does not yet have an approved label even if there are promising findings both in preclinical and early-clinical settings [25]. Current clinical trials are evaluating the safety and efficacy of treatments combining Olaparib, Niraparib, Rucaparib and RT in several settings but there are no data to this date.

## 5. Discussion

To our knowledge, this is the first review specifically addressing the topic of concomitant administration of PARP-I during photon beam RT. Concerning the key questions examined in the current analysis according to the PICO approach (Table 1), it emerges that: (Queries 1 and 2) the combination of PARP-I and RT is feasible and safe, with a range in terms of survival and local control that it is different in the series according to the histology. When a comparison was available, no distinct differences in survival rates, as well as local control across the arms, were achieved. The most common severe toxicities (Query 2) were hematological in accordance to literature data about PARP-I in which these AEs were the frequent reason for dose modification, interruption or discontinuation [26]; with regards to non-hematological toxicities, gastrointestinal AEs (diarrhea, nausea, colitis and enteritis) were the most recurring as well as fatigue that seemed to be a class effect as reported by LaFargue et al. [26]. Except for the series by de Hann et al. [22] in which the triple combination of RT, low-dose daily CDDP and Olaparib (25 mg once or twice daily) was not tolerable because of esophageal and hematologic toxicity, no significant differences were observed in the other three studies in which comparison is usable, in terms of adverse events across treatment arms. It is understandable that in a triple combination, if there is not a comparison arm with a double combination, it is difficult to understand which treatment (RT or chemotherapy) contributes most to the observed toxicities. Indeed, several clinical trials testing the combination of PARP-I with different drugs, without concomitant RT, reported significant toxicities that led to trial discontinuation or discouraged future studies [26]. It should consequently be stressed the prime importance of an accurate analysis and an exhaustive comprehension of the toxicities in each setting in order to guarantee a safe and effective treatment for the patients.

In the current analysis, the combination of RT with PARP-I appeared safe in the included trials, but these data currently do not support their administration in the analyzed population, since three series concluded for a lack of addition clinical benefit by adding PARP-I.

Moreover, 10 studies concluded for a recommended DLT [12,14,16,17,18,19,20,21,22,23] but Sim et al. [24], as well as Chabot et al. [15], reported a lack of clinical benefit. Moreover, Baxter concluded for a lack of clinical benefit compared with contemporary historical series [21]. The lowest dose level with CDDP was above the MTD because of hematologic and late esophageal DLT in the trial by de Hann et al., and the corresponding MTD without cisplatin was Olaparib 25 mg once daily [22].

The limitations of the studies included in the current analysis do not allow fully definitive conclusions especially because of the heterogeneity of evaluated tumor types and patient populations; in addition, most of the clinical studies lacked comparison arms. Moreover, Veliparib, the main PARP-I reviewed, does not yet have an approved label even if there are promising findings both in preclinical and early clinical settings [25]. Olaparib, Niraparib, Rucaparib and Talazoparib [27] are currently tested in combination with RT in ongoing clinical trials and thus far there are 29 ongoing clinical trials that have been registered at clinicaltrials.gov (accessed on 28 September 2021). Waiting for these expected results and considering the above-reported bias, some observations might be highlighted.

Patient-, cancer- and treatment-related factors (especially RT localization and doses) are of pivotal importance to propose PARP-I during RT.

The current analysis included 823 patients with different histology and clinical settings (brain metastases, glioblastomas, NSCLC, breast cancers, gynecological tumors, pancreatic cancers, rectal cancers, head and neck and pediatric pontine gliomas). It appears glaring that the toxicity-endpoint of the current analysis should be interpreted with caution due to heterogeneity of RT volumes, fields, fractionation schemes and total doses that are crucial points in the evaluation of local toxicities.

Despite this limitation, to be added to the small cohorts and the lack of data from randomized controlled trials, it was difficult to draw definitive conclusions about the tolerability in each clinical setting; the current data show the tolerability of combination in RT + PARP-I.

Considering the data by de Haan et al. [22] and in light of that Olaparib is replication dependent [28,29] and the worse toxicities resulting in rapidly proliferation tissues, further studies on triple combination approach should pay special attention to patient selection and RT techniques/fractionation schedules [30].

Concerning cancer and treatment hallmarks, PARP- I proved to be a radiosensitizer both in cell lines and xenograft models due to the synergic effects in DNA damage caused by ionizing radiation and the inhibition of proteins essential for DNA damage repair by PARP-I. They inhibit tumor cell proliferation, decrease clonogenicity survival, set back tumor growth as well as improve survival in mice [31]. In a recent systematic review of literature, PARP-I proved to be an efficient radiosensitizers capable of enhanced death ratio between 1.04 and 2.87 in several tumor models [31,32].

Although PARP-I improved the antitumor effect to RT, they also boosted the RT response in replicating normal tissues [30]. Considering the physical characteristics of particle beam RT and due to the above-mentioned effects of PARP-I with RT, a combination approach with hadrons, and in particular, carbon ions (that are able to determine clustered DNA damages that are unlikely recoverable by the cellular repair mechanism) [33] should be investigated. Indeed, remarkable in vitro findings are reported in recent literature about the interaction between PARP-I and particle beam RT. Hirai et al. [34] demonstrated the radiosensitization effect of PARP-I in human pancreatic MIA PaCa-2 cancer cell line treated with carbon ion radiation therapy (CIRT) probably related to the switch of sublethal oxidative clustered DNA lesions (OCDL) to fatal ones via BER pathway. Moreover, PARP-I also proved to magnify the effect of hadrons on chondrosarcoma cells [35].

Considering the lack of data in the literature and the absence of significant evidence of the combination approach (RT and PARP-I), other points need to be highlighted: (1) is there a correct interval of administration of PARP-I during RT (sandwich vs. sequential), considering also the radiosensitizer efficacy? (2) In the sandwich approach, what is the optimal interval between RT and PARP-I administration? Indeed, it is relatively common in clinical practice to stop PARP-I during RT, considering their radiosensitizer effects, to avoid severe AEs, but the correct interval taking into account a risk/benefit ratio is still not clear.

## 6. Conclusions

Notwithstanding the limitations of the analyzed studies, the available data here discussed for the combination between photon beam RT and PARP-I, allow us to conclude that this approach is feasible and usually safe, with hematological toxicities being the most commonly represented AEs. The data about efficacy could not accurately be determined because of the heterogeneity of data (related to patient and tumor types). However, considering the radiosensitizer action of PARP-I, toxicity should not be underestimated and a correct selection of patients fit for a combination approach should be warranted. The optimization of patient selection, RT techniques/dose/fractionation and PARP-I dose/timing in order to lessen the normal tissue response and improve anti-tumor efficacy remains a major challenge. Future studies focusing on clinical outcomes and aiming to assess the optimal schedule, the optimal time of potential suspension or PARP-I dose titration in combination with modern RT techniques, such as particle RT, are advocated.

## Figures and Tables

**Table 1:** Research questions according to PICO criteria.

Query	Population	Intervention	Comparison	Outcomes
1	Patient with new diagnosis or recurrent tumor	Radiotherapy and concomitant Parp-I	Radiotherapy alone or with chemotherapy or standard of care (if available)	Locoregional control Disease free survival Overall survival
2	Patient with new diagnosis or recurrent tumor	Radiotherapy and concomitant Parp-I	Radiotherapy alone or with chemotherapy or standard of care (if available)	Acute and late toxicity with grade ≥ 3

**Table 2:** Key characteristics and description of selected studies.

Study		Population			Intervention			
Author	Type of Study	Number of Patients (pts)	Age Median [Range]	Localization	Radiotherapy (Total Dose/Fraction) with or without Chemotherapy	Aim of Radiotherapy	Parp-INHIBITORS	Follow Up
Mehta (2015) [14]	Single-arm dose-escalation phase I	81	58 (31–84)	Brain metastases	30 Gy/10 fr or 37.5 Gy/15 fr	Definitive	Veliparib	NA
Chabot (2017) [15]	Three-arm phase II controlled trial; placebo (102 pts) vs. Veliparib 50 mg (103 pts) vs. Veliparib 200 mg (102 pts).	307	60 (41–86) (placebo arm) vs. 60 (33–83) (50 mg BID arm) vs. 62 (39–81) (200 mg BID arm)	Brain metastases	30 Gy/10 fr	Definitive	Veliparib	NA
Czito (2017) [16]	Single-arm dose-escalation phase I	32	57 (37–75)	Rectum	50.4 Gy/28 fr + capecitabine 825 mg/m^2^ twice daily	Neoadjuvant	Veliparib	NA
Reiss (2017) [17]	Single-arm dose-escalation phase I	32	58 (55–65)	Peritoneal carcinomatosis (ovarian and fallopian cancer)	21.6 Gy/36 fr (BID)	Radical	Veliparib	45 months
Jagsi (2018) [18]	Single-arm dose-escalation phase I	30	50.5 (41–40)	Breast (inflammatory or locoregionally recurrent)	50 Gy + 10 Gy (boost)/25 fr	Adjuvant	Veliparib	3 years
Karam (2018) [19]	Single-arm dose-escalation phase I	16	61 (46–75)	Head and Neck (locally advanced)	69.3 Gy/33 fr + Cetuximab	Radical	Olaparib	26 months
Tuli (2019) [20]	Single-arm dose-escalation phase I	30	68 (60–77)	Pancreas (locally advanced)	36 Gy/15 fr + gemcitabine 400 mg/m^2^	Radical	Veliparib	NA
Baxter (2020) [21]	Phase I/II trial: single-arm dose-escalation trial with comparison to historical series	66	6.6 (2.2–15.8)	Diffuse intrinsic pontine glioma	54 Gy/30 fr + Temozolomide (135 mg/m^2^ d1–5/28 d)	Radical	Veliparib	6.3 months (stopped early for futility)
de Haan (2020) [22]	Phase I: two parallel arms dose-escalation trial	28	58 (55–65) (Arm CDDP+ Olaparib) 62 (58–68) (Arm Olaparib)	Lung (NSCLC)	66 Gy/24 fr (1 pts s stopped at 52 Gy) CDDP 6 mg/m^2^/daily in the CDDP + Olaparib arm	Radical	Olaparib	14 months
Argiris (2021) [23]	Single-arm dose-escalation phase I trial follow by a two-arm controlled phase II trial placebo (13 pts) vs. Veliparib (18 pts)	21 (phase I) and 31 (phase II)	phase I: 70 (53–81). Phase II: 64.7 (47–78.9) (arm Veliparib), 65 (56.6–75.6) (arm placebo)	Lung (stage III NSCLC)	60 Gy/30 fr + paclitaxel 45 mg/m^2^/carboplatin AUC2 (weekly concomitant and in consolidation)	Radical	Veliparib (concomitant and consolidation)	Phase I: 40.6 months; phase II: 26.9 months
Sim (2021) [24]	Two-arm controlled phase II trial; standard arm(41 pts) vs. Veliparib 200 mg (84 pts)	125	60 (22–78) (Veliparib arm) vs. 62 (24–73) (Standard Arm)	Glioblastoma (unmethylated MGMT promoter)	60 Gy/30 fr + Temozolomide (75 mg/m^2^ OD concomitant and 150–200 mg/m^2^ d1–5/28 d)	Radical	Veliparib	27.2 months
Loap (2020 and 2021) [12,13]	Single-arm dose-escalation phase I	24	46 (25–74)	Breast (triple negative)	50 Gy/25 fr; 50.4 Gy/28 fr ± SIB tumor boost (63 Gy)	Adjuvant	Olaparib	12 months

**Table 3:** Double and Triple combination outcomes.

**a. Double Combination Outcomes**					
**Study**	**Toxicity ≥ Grade 3**	**Main ≥ Grade 3 Hematological Toxicities**		**Main ≥ Grade 3 Non-Hematological Toxicities**	**Clinical Outcome**
Mehta (2015) [14]	29.6% (acute)	lymphopenia (5%), anemia (4%)		Fatigue (7%) hyponatremia (6%) dehydration (5%) hyperglycemia (4%)	6-month OS: 54%
Chabot (2017) [15]	43% (acute, placebo arm) vs. 28% (50 mg BID arm) vs. 25% (200 mg BID arm)	anemia (3% vs. 1% vs. 2%, for placebo, 50 mg BID and 200 mg BID, resp.); thrombocytopenia (1% vs. 3% vs. 2%).		pulmonary embolism (1% vs. 4% vs. 2% for placebo, 50 mg BID and 200 mg BID resp.); pneumonia (8% vs. 3% vs. 2%) fatigue (4% vs. 2%. vs. 2%) hyperglycemia (1% vs. 2%. vs. 2%)	OS: 185 d. (placebo) vs. 209 d. (50 mg) vs. 209 d. (200 mg) ORR: 41.2% (placebo) vs. 36.9% (50 mg) vs. 42.2% (200 mg)
Reiss (2017) [17]	≥59% (acute)	lymphopenia (59%), thrombocytopenia (12%), anemia (9%), neutropenia (6%)		nausea (6%) diarrhea (6%) anorexia (6%) vomiting (6%) fatigue (6%)	Median PFS: 3.6 months. Median OS: 9.1 months
Jagsi (2018) [18]	46.7% (late; at year 3)	no late G3 hematological toxicity		fibrosis (40% at 3 years) lymphoedema (20%) skin induration (13%) chest wall pain (7%) atrial clot (7%)	3-year disease control: 50% (15 failures) 3-year OS: 56.7% (13/30 deaths).
Loap (2020 and 2021) [12,13]	Acute toxicity ≥50% Late (1-year) toxicity: 0%	Acute: lymphopenia (50%) Late: 0% treatment related		Acute: Radiodermatitis (8%) pain (4%) Late 0% treatment related	1-year OS 96% (88%-100%)
**b. Triple combination outcomes**					
**Study**	**Drug**	**Toxicity ≥ Grade 3**	**Main ≥ Grade 3** **Hematological Toxicities**	**Main ≥ Grade 3** **Non-Hematological Toxicities**	**Clinical Outcome**
Czito (2017) [16]	capecitabine 825 mg/m^2^ twice daily	25% (acute)	anemia (3%), lymphopenia (3%)	diarrhea (9%) radiation skin injury (3%) hyperglycemia (3%) pulmonary embolism (3%) syncope (3%) vaginal stricture (3%) radiation enteritis (3%)	pCR: 29%. Tumor downstaging: 71%. CEA response rate: 68%
Karam (2018) [19]	Cetuximab 400 mg/m^2^ (5–7 day before RT) and 250 mg/m^2^ weekly	≥69% (acute)	lymphopenia (19%)	mucositis (69%) dermatitis (38%) dysphagia (31%) nausea (13%) dehydration (13%) hypomagnesemia (13%) malnutrition (13%) vomiting (13%) oral pain (6%) weight loss (6%)	2-year OS: 72% 2-year PFS: 63% 2-year LC: 72% 2-year DC: 79%
Tuli (2021) [20]	Gemcitabine 400 mg/m^2^	>96% (acute)	lymphopenia (96%) anemia (38%) thrombocytopenia (19%) neutropenia (4%) febrile neutropenia (4%)	anorexia (19%) abdominal pain (12%) nausea (12%) vomiting (8%) diarrhea (4%) colitis (4%) fatigue (4%)	median PFS: 9.8 months; median OS: 14.6 months
de Haan (2020) [22]	CDDP 6 mg/m^2^/daily	80% in CDDP + Olaparib arm vs. 57% Olaparib BID vs. 45% Olaparib once/day	CDDP + Olaparib arm: Neutropenia (30%, Lymphocytopenia (70% G3, 30% G4 with 1% G3 late), Thrombocytopenia G4 (10%) Olaparib BID Lymphocytopenia (23% G3, 14% G4 with 17% G3 late) Olaparib once daily: Lymphocytopenia (18% G3, 18% G4 with 11% G3 late)	CDDP + Olaparib arm: Gastro-intestinal (33%) G4 fibrosis (11%), G5 fibrosis (11%) Olaparib BID: gastrointestinal (29% acute,17% late) Pneumonitis (17%) Lung hemorrhage G4 (17%) Olaparib once daily: gastrointestinal (9% acute only). Lung infection (9%), dyspnea (9%), pneumonitis G5 (9%), lung hemorrhage G5 (11%) and fibrosis G5 (11%); spinal fracture (11%)	2-year LC:84% (with a 95% confidence interval of 58–95%; 89% with cisplatin and 83% without cisplatin Median PFS: 6.5 months (oligometastatic) and 12 months (Locally advanced) Median OS: 23 months (oligometastatic) and 28 months (locally advanced)
Baxter (2020) [21]	Temozolomide (135 mg/m^2^ d1–5/28 d)	≥50% (acute)	lymphopenia (50%) neutropenia (32.7%) thrombocytopenia (23.1%)	maculopapular rash (3%) neurological deterioration (2%)	1-year OS: 37.2% 2-year OS: 5.3% PR: 14%
Argiris (2021) [23]	Paclitaxel 45 mg/m^2^/carboplatin AUC2 (weekly concomitant and in consolidation)	Phase I: 81% (acute), including one treatment-related G5 esophageal perforation. Phase II: 47% (arm Veliparib); 69% (arm placebo)	Phase I: lymphopenia (57%); neutropenia (38%); Phase II: neutropenia (18%), thrombocytopenia (3%)	Phase I: esophagitis (19%), fatigue (10%); esophageal perforation (5%) Phase II: anorexia (6%), esophageal pain (6%), fatigue (6%), hyperglycemia (6%), oral mucositis (1%)	1-year PFS: 43% (Veliparib) vs. 40% (placebo); 1-year OS: 76% (Veliparib) vs. 50% (placebo)
Sim (2021) [24]	Temozolomide (75 mg/m^2^ OD concomitant and 150–200 mg/m^2^ d1–5/28 d)	55% (both Veliparib and standard arm)	Veliparib arm: thrombocytopenia (17%); neutropenia (12%). Standard arm: thrombocytopenia (8%), neutropenia (3%)	Veliparib arm: seizures (11%), fatigue (7%). Standard arm: seizures (5%), hyperglycemia (5%), diarrhea (5%)	median PFS: 5.7 months (Veliparib) vs. 4.2 months (standard); median OS: 12.7 months (Veliparib) vs. 12.8 months (standard)

Legend Table 3a,b: for each analyzed study, the percentages are referred to all the patients included.
